# Cyclotide Structure–Activity Relationships: Qualitative and Quantitative Approaches Linking Cytotoxic and Anthelmintic Activity to the Clustering of Physicochemical Forces

**DOI:** 10.1371/journal.pone.0091430

**Published:** 2014-03-28

**Authors:** Sungkyu Park, Adam A. Strömstedt, Ulf Göransson

**Affiliations:** Division of Pharmacognosy, Department of Medicinal Chemistry, Uppsala University, Uppsala, Sweden; University of Edinburgh, United Kingdom

## Abstract

Cyclotides are a family of plant-derived proteins that are characterized by a cyclic backbone and a knotted disulfide topology. Their cyclic cystine knot (CCK) motif makes them exceptionally resistant to thermal, chemical, and enzymatic degradation. Cyclotides exert much of their biological activity via interactions with cell membranes. In this work, we qualitatively and quantitatively analyze the cytotoxic and anthelmintic membrane activities of cyclotides. The qualitative and quantitative models describe the potency of cyclotides using four simple physicochemical terms relevant to membrane contact. Specifically, surface areas of the cyclotides representing lipophilic and hydrogen bond donating properties were quantified and their distribution across the molecular surface was determined. The resulting quantitative structure-activity relation (QSAR) models suggest that the activity of the cyclotides is proportional to their lipophilic and positively charged surface areas, provided that the distribution of these surfaces is asymmetric. In addition, we qualitatively analyzed the physicochemical differences between the various cyclotide subfamilies and their effects on the cyclotides' orientation on the membrane and membrane activity.

## Introduction

**Figure 1:**
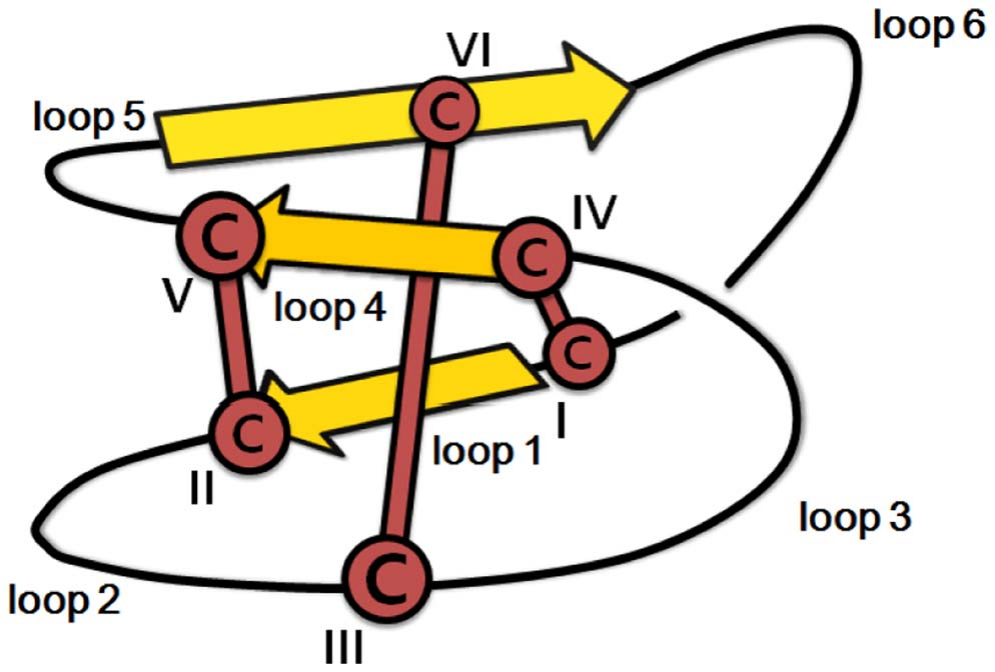
Schematic illustration of cyclotide structure. A 3D illustration of the cyclotide structure showing the CCK motif (with cysteines labeled I–IV), and the inter-cysteine loops (labeled 1–6). The CCK motif is characterized by a cyclic peptide backbone and the knotted disulfide bonds (I–IV, II–V, III–VI), which are indicated by thick brown bars. The structure usually contains antiparallel beta strands (indicated by large yellow arrows) and several tight turns.

The cyclotides are a family of proteins characterized by their cyclic backbone and knotted disulfide topology (Figure 1) [1], [2]. The cyclic cystine knot (CCK) motif makes the cyclotides exceptionally resistant to thermal, chemical, and enzymatic degradation [3]. However, it also means that their structure is “inside out” compared to that of most other globular peptides and proteins: their hydrophobic residues are forced onto their external surface because the inner core is occupied by the cystines. Currently, cyclotides have been found in limited set of plant families, namely the Rubiaceae (coffee) [4], the Violaceae (violet) [5], the Fabaceae (legume) [6] and the Solanaceae (potato) families [7]. In addition, peptides with similar structures and sequences have been found in the Poaceae (grass) [8] and Cucurbitaceae (gourd) [9] families. Sequence variation is immense: it has been estimated that the number of different cyclotides in Rubiaceae alone exceeds 50,000 [4]. Cyclotides form a combinational peptide library in those plant species that express them, and their function appears to be related to plant defense as reflected in their potent insecticidal [10] and antimicrobial activity [11], [12]. Cyclotides also have pharmaceutically relevant properties including anti-cancer [13] and anti-HIV activity [14], and have proven to be good scaffolds for protein engineering [15]. These features make cyclotides potentially valuable peptides for pharmaceutical and agrochemical applications.

The known cyclotides have been divided into two main subfamilies based on the presence or absence of a conceptual 180° twist in the cyclic backbone caused by a conserved *cis*-Pro residue in loop 5, which lies between two cysteine residues [1]. Cyclotides that contain this twist are referred to as Möbius cyclotides; those without it are referred to as bracelet cyclotides. Some loops (loops 1 and 4) have high sequence similarity between the subfamilies while others (loops 2 and 3) are conserved only within individual subfamilies. Recent studies [16] have described hybrid cyclotides that exhibit sequence characteristics of both the Möbius and bracelet subfamilies. A third minor subfamily, known as the trypsin inhibitors, has been discovered in gourd plants. These peptides contain the CCK motif but do not otherwise exhibit any sequence homology with the other subfamilies [9].

Although the cyclotides exhibit diverse biological effects, there is a growing body of evidence suggesting that most of these activities are due to their ability to interact with and disrupt cell membranes [17], [18], [19], [20]. This ability arises from a combination of hydrophobic and electrostatic interactions between the membrane and the cyclotide. In particular, exposed hydrophobic and electrostatic patches on the surface of the peptide contribute to its binding and dictate the orientation of its binding with respect to the CCK-motif [21]. The integrity of the hydrophobic patch is important for the cyclotides' biological activity, as demonstrated by experiments using chemically modified analogues [18], [22] and an alanine scan [19].

Although the extremely stable and rigid cyclotide structure appears ideal for structure-activity relationship (SAR) analyses, no attempt has yet been made to establish quantitative SARs (QSARs) for these peptides. Several authors have presented QSAR models for other membrane-active peptides, especially antimicrobial peptides [23]. Like cyclotides, these peptides are thought to exert their bioactivities via membrane disruption and/or lysis. Some of these studies [24], [25] included variables that represent the conformational energy or the shape of the folded peptide in their model equations, probably because the studied proteins are quite flexible. However, this approach does not produce models that allow for easy geometrical interpretation or provide guidelines for the design of more active compounds. Another potential problem in QSAR modeling is the overfitting of data due to the inclusion of multiple variables that describe similar physicochemical properties. While this can yield models with very high regression coefficients, they can also be easily misinterpreted [23], [25]. Recently, Frecer [26] developed a method that classifies the positions of amino acid residues within a peptide based on their orientations and physiochemical properties, but this method appears to be limited to the analysis of minor changes in peptide sequences.

In this work, we present a method for qualitatively and quantitatively characterizing the lipophilic and electrostatic properties of cyclotides using only four variables. We use this method to classify cyclotides, and to build a QSAR model linking those physicochemical properties to their cytotoxic and anthelmintic activities. In contrast to previous SAR/QSAR studies on peptides of similar size and activity, our approach accounts for both the total extent of the molecular surface area that exhibits a given physicochemical property and also the orientation and distribution of those surfaces.

## Materials and Methods

### Template selection

MUSCLE [27] was used to perform a multiple sequence alignment of each target sequence against 13 cyclotides whose structures have been solved by NMR: kalata B1 (pdb code: 1nb1) [28], kalataB2 (1pt4) [29], kalata B8 (2b38) [16], [W19K,P20N,V21K]-kalata B1 (2f2j) [30], [P20D,V21K]-kalata B1 (2f2i) [30], circulin A (1bh4) [31], circulin B (2eri) [32], cycloviolacin O1 (1nbj) [28], cycloviolacin O2 (2knm) [33], cycloviolacin O14 (2gj0) [34], varv F (2k7g) [35], vhr1 (1vb8) [36] and tricyclon A (1yp8) [37]. Optimized alignments were created by adjusting the initial alignments generated using MUSCLE with respect to conserved residues. A Neighbor Joining (NJ) tree [27] was constructed based on the multiple sequence alignment, using nonparametric bootstrapping with 1000 replicates. The cyclotide closest to the target sequence in the resulting tree was then used as a template for molecular modeling. All of these phylogenetic analyses, including the MUSCLE sequence alignment and the construction of the NJ tree, were performed using version 4.03 of the SeaView software package [27]. The template used for the linear cyclotide psyle C was violacin A (2fqa) [38]. The first conformation of the NMR ensemble reported in the PDB was used as template and, when applicable, for calculation of molecular descriptors.

### Structure construction

Structures were constructed based on the pairwise sequence alignments of each target sequence with the selected templates using Modeller 9v8 [39]. Twenty structures were generated for each sequence, and the modeled structures were evaluated using the DOPE potential and the GA341 score as implemented in Modeller 9v8. Of the structures whose GA341 scores were closest to 1 (indicating a native-like fold), the three with the lowest DOPE potentials were selected for structure refinement. In cases where two templates were chosen for one target sequence, six structures were refined.

### Refinement of models

The selected structures were assigned appropriate ionization states using the Protonate3D module of the MOE package and then refined by performing conformational searches with LowMode MD [40] using the AMBER94 force field and Generalized Born solvation (GB). The root mean square deviation (RMSD) gradient was set at 0.5 Å and the RMSD limit at 0.75 Å, with a maximum of 10000 iterations of energy minimization and a rejection limit of 100. In cases where the refinement of the overall structure failed for all of the conformations imported from Modeller 9v8, a few loops were selected and refined individually with LowMode MD. Predicted structures established through conformational searches covering the entire peptide structure were accepted if the RMSD value for the Cα atoms was less than 2.0 Å (relative to their positions in the initial conformation) and the Φ and ψ angles of the predicted structure were within the allowable regions of the Ramachandran plot. In cases where conformational searches covering the entire peptide structure did not produce acceptable results, constrained searches were performed in which only one or two loops were allowed to vary their conformations. Loops were selected for constrained optimization if they contained residues whose side chains had different physicochemical properties relative to their counterparts in the template loop, or if they contained multiple gap regions compared to the template loop. The backbones of the unselected loops and the cystine residues were constrained by fixing their atomic positions, and the side-chains of these fixed loops were subjected to low frequency mode vibration. Loop conformations were then sampled and ranked according to their potential energy.

### Calculation of molecular descriptors

Descriptors were calculated using scientific vector language (SVL) as implemented in the Molecular Operating Environment (MOE) [41]. These descriptors include lipophilic moment (L_M_), exclusive lipophilicity 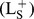, total lipophilicity (L_S_), Hydrogen Bond Donor (HBD) surface area (E_S_) and HBD amphipathic moment (E_M_).

### Generation of a lipophilicity scale and the calculation of solvent-accessible areas

The Molinspiration Property Calculation Service [42] was used to predict the lipophilicity of amino acid side chains. The amino acids' logP values were used as the starting point for these calculations. The logP values were parameterized, scaled, and normalized with respect to glycine. The maximum solvent accessible surface area (SASA) of the side chain of each amino acid (X) was then calculated based on the structure of the tripeptide Gly-X-Gly in an extended conformation (Φ = ψ = 180°) [43]. For natural amino acids (X), the tripeptide was constructed using data from rotamer libraries. For tripeptides that contained a non-natural amino acid, the appropriate structural modification was introduced into the relevant precursor amino acid using the builder module implemented in MOE, and the conformation of the modified side chain was relaxed using the MFF94x force field with GB solvation.

### Statistical analysis

Materials studio 6.0 [44] was used to generate all QSAR models. Minitab 16 [45] was used to transform the molecular descriptor data.

### Hierarchical clustering analysis

Ward's reciprocal nearest neighbor method was used to perform a hierarchical cluster analysis of 75 cyclotides. Distances were measured in Euclidean terms, using the four molecular descriptors E_M_, E_S_, L_M_ and 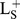 as variables.

## Results and Discussion

The lipophilic and electrostatic properties of cyclotides determine how they adsorb to membranes and the mechanisms by which they disrupt membrane integrity [46]. Here we describe a QSAR model that uses four molecular descriptors representing the lipophilic and electrostatic properties of cyclotides in terms of their surface area - a scalar quantity - and their orientation relative to the protein surface (the moment). We show that these descriptors are useful for characterizing and classifying cyclotides, and for understanding their structure-activity relationships. To this end, we analyze the physicochemical properties of the 68 known cyclotides with cytotoxic activity [13], [18], [22], [47], [48], [49], [50] against the lymphoma cell line U937-GTB or anthelmintic activity [20], [51] against the caterpillar larvae of *H. contortus*.

### Cyclotide homology modeling

**Figure 2:**
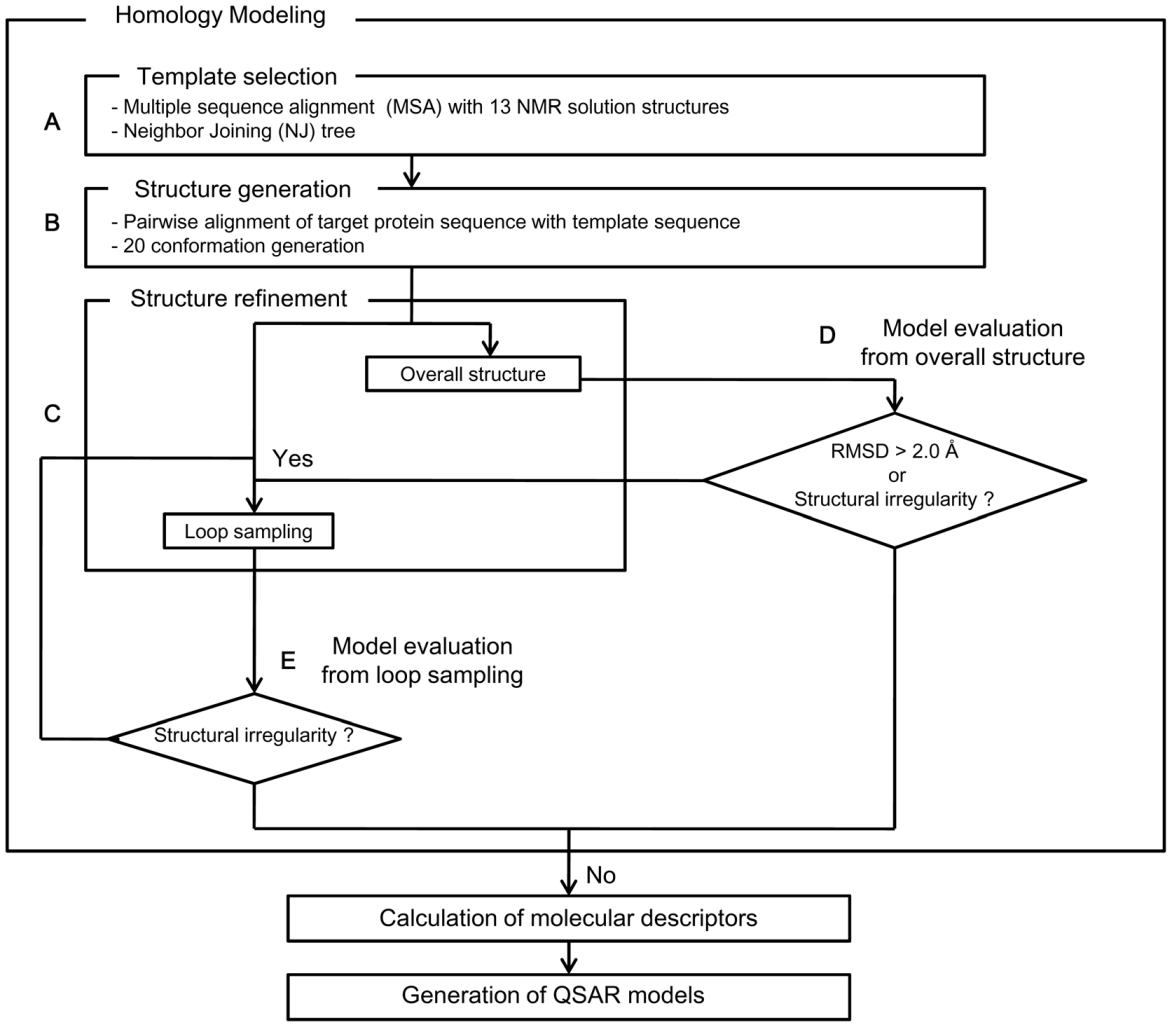
Flow chart depicting the methodology used for QSAR modeling. Predicted structures for the cyclotides were obtained through homology modeling using the sequences of the 13 cyclotides whose solution-phase structures had previously been determined by NMR. A) To identify appropriate templates, each target sequence was aligned with those of the 13 peptides with known structures. A neighbor joining (NJ) tree was then constructed based on the multiple sequence alignment. The resulting cladogram was used to identify the closest relatives of the target sequence with a known structure, which were then used as templates for modeling the unknown structure of the target peptide. B) For each template sequence, 20 PDB structures were generated based on the pairwise sequence alignment of the target sequence with the selected templates. These 20 conformations were evaluated using the DOPE potential and GA341 score, and the best three conformations were selected. If two templates were chosen for a target sequence, a total of 6 PDB structures were selected. C), D) and E) The structures generated during step B were subjected to conformation searches. If structural refinement of the protein as a whole yielded a result with an unacceptable geometry or one for which the RMSD of the Cα-atoms was greater than 2.0 Å relative to the starting conformation, two loops were selected for loop sampling. Loops were selected based on their sequences, focusing on those containing residues with different physicochemical properties relative to those in the corresponding positions of the template sequence or those that aligned with gaps in the template sequence. After structure refinement, one conformation of each peptide sequence was selected for use in calculating the molecular descriptors. The molecular descriptors of the cyclotides, together with their activity data were used as variables in QSAR modeling.

We used cyclotide structures reported in the Protein Data Bank as solved by NMR, when possible. However, the majority of structures had to be constructed by homology modeling. Molecular properties of cyclotides were calculated based on the lowest energy conformation that was identified for each one. These low-energy conformers were identified through a homology modeling process involving template selection followed by structure generation and refinement (Figure 2).

**Figure 3:**
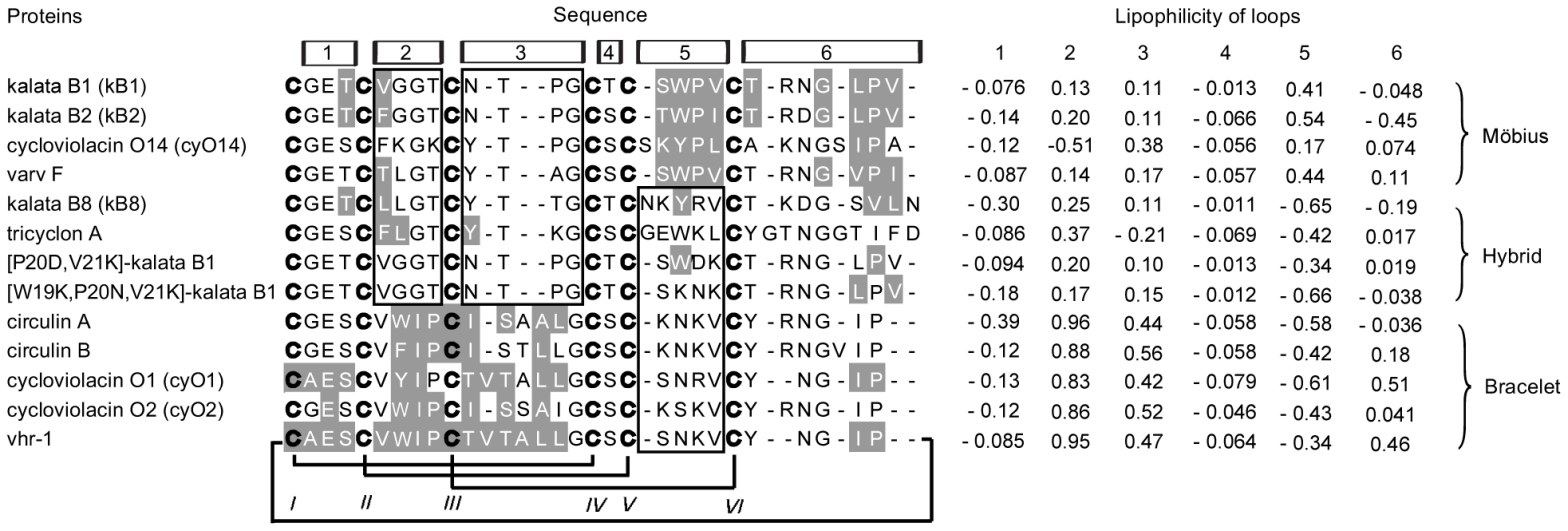
The sequences of the membrane-buried regions of selected cyclotides and their lipophilicity profiles. The multiple sequence alignment shows all of the cyclotides for which 3D structures (based on solution-phase NMR studies) are available. These sequences were used as template candidates for homology modeling of the cyclotides of unknown structure. The cysteine of loop 1 was arbitrarily selected as the N-terminus for the multiple sequence alignments. The residues required for cyclization, i.e. the conserved C-terminal residue (Asn or Asp) and N-terminal residue (Gly), are positioned within loop 6. The membrane-buried residues are highlighted with a gray background. OPM was used to predict the orientation of the selected cyclotides on the membrane. Among the members of the Möbius subfamily, many of the loop 5 and 6 residues are membrane-buried. Among the bracelet cyclotides, large proportions of loops 2 and 3 are buried. Only a couple of residues from the cyclotides of the hybrid subfamily penetrate the membrane interface. The lipophilicity of the loops was calculated as the total lipophilicity (L_S_) of all of the residues within the loop. It should be noted that the membrane-buried regions identified by OPM are consistent with those exhibiting high lipophilicity.

**Figure 4:**
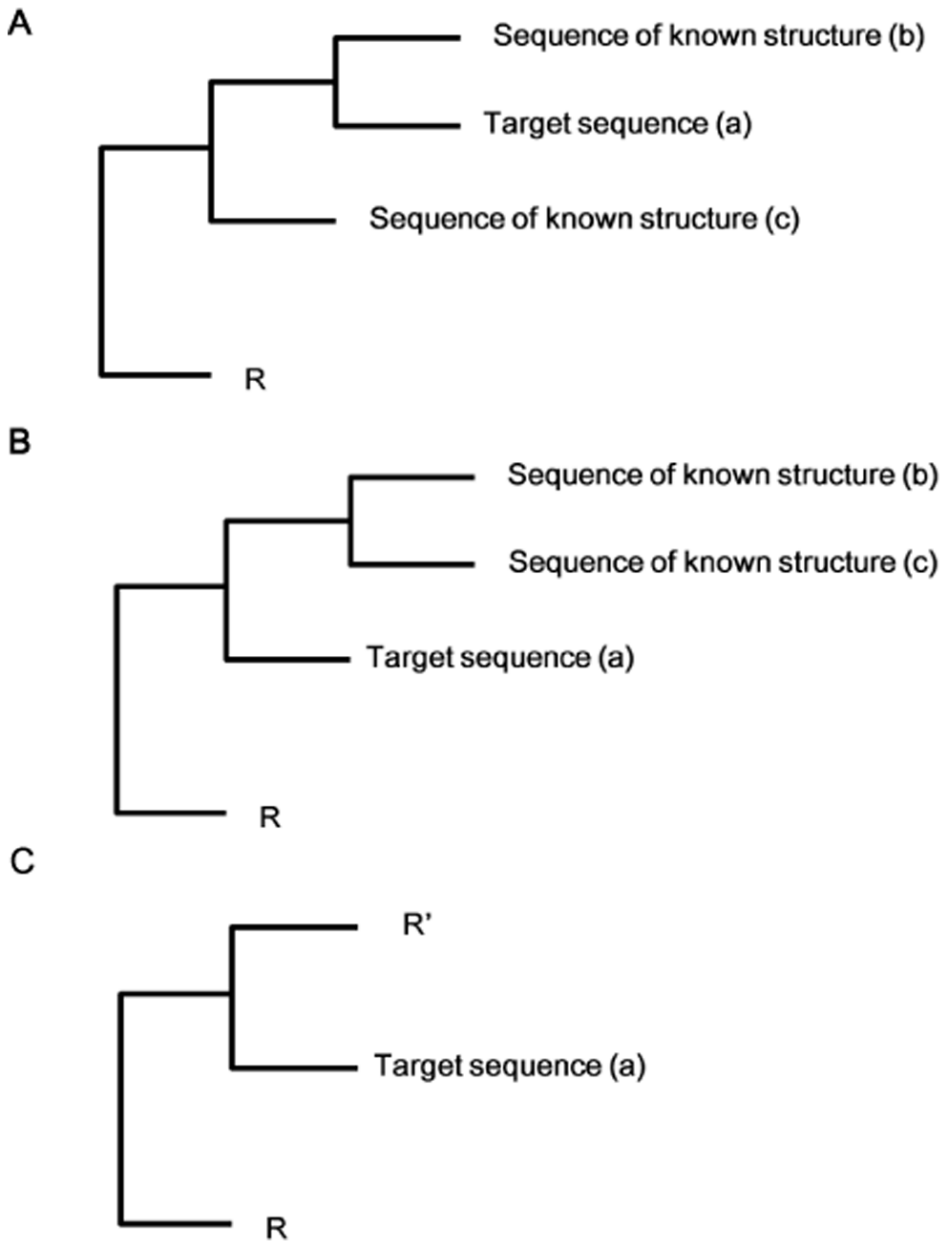
Template selection strategy based on the NJ tree. This figure illustrates the strategy of a template selection using a cladogram in which the target sequence (a) relates differently to various sequences of known structure. The symbols R and R′ represent the other branches of the cladograms. These branches contain sequences of known structure. Our aim was to identify sequences of known structure that could reasonably be used as templates for the target sequence based on the bootstrapped consensus tree. The procedure used to select the template sequences most closely related to the target sequence from the bootstrap NJ tree depended on the structure of the tree and the position of the target sequence relative to the nearest neighbors of known structure. In cases such as that shown in subfigure A, a single peptide of known structure (b) was selected as the template for target sequence (a) if the bootstrap value for the two taxa, a and b, was greater than 50%. Two taxa, b and c were selected if the bootstrap value for taxa a and b was below 50%. In cases such as that shown in subfigure B, two taxa, b and c were selected as templates, regardless of the bootstrap value of the common ancestor of the three taxa, a, b and c. In case C, we randomly chose two templates from the sister taxa (R′) of the target sequence (a) if the sister taxa (R′) included two or more sequences of known structure.

Traditionally, template selection is done using either protein threading [52], [53] or database searches using BLAST [54] or FASTA [55]. Unfortunately, none of these methods are well suited to cyclotides because of their high sequence similarity. To find the most appropriate template for each cyclotide from the 13 cyclotide NMR structures available in the Protein Data Bank (PDB), we used a neighbor joining (NJ) tree to describe the homology of the cyclotides based on sequence similarity. As shown in Figure 3, the first cysteine residue on the N-terminal side of loop 1 was arbitrarily defined as the N-terminus of each peptide. Multiple sequence alignments were performed using MUSCLE and then corrected manually. Manual corrections were performed to ensure that, among other things, all of the cysteine residues were kept in fixed positions, as well as the N-and C-terminal residues of loop 6, which must be conserved to enable circularization. Based on their bootstrap values, one template (or two in some cases) was selected for each sequence of unknown structure (Figure 4).

Modeller was then used to generate candidate structures, which were refined using LowMode MD [29] to identify the lowest energy conformation of each cyclotide. To avoid the pitfall of local energy traps, which can stop the refinement process once the first stable conformation is encountered, any refined structure for which the RMSD value of the Cα atoms was greater than 2.0 Å relative to the template structure was regarded as a decoy. The refinement process was conducted until a global minimum structure was located that had an RMSD value of less than 2.0 Å and which satisfied the requirements of protein geometry [56]. It is adequate to use one conformation only to represent the structure of cyclotides in solution as judged from experimentally determined NMR ensembles, at least for the calculation of molecular descriptors. For typical cyclotides, the average RMSD values of peptide backbone and side chains of the NMR ensemble do not exceed 2.0 Å, and the exposure of side chains at the surface is more or less constant (Figure S1).

### Design and calculation of molecular descriptors

The cyclotides were characterized using descriptors of the lipophilicity and electrostatic properties that are important in peptide-membrane interactions. We associate each such physicochemical property with a scalar value and a moment. The scalar value quantifies the molecular property without providing any information concerning its distribution on the molecular surface. For example, the surface area scalar quantifies the extent of the molecular surface that exhibits a certain property. Being scalar quantities, surface areas can be summed without regard for their orientation or position on the molecular surface. However, their distribution can also be described using a vector whose direction reflects the relative orientation of the property on the molecular surface. The moment, i.e. the length of the vector, is proportional to the degree of asymmetry in the distribution of the physicochemical property of interest across the molecular surface.

#### A lipophilicity scale for natural and non-standard amino acids

Because some of the cyclotides examined in this work contain chemically modified amino acids, it was necessary to create a lipophilicity scale that can describe such residues. The lipophilicity of the side chains was therefore predicted using their logP values (Table 1). To validate the lipophilicity values for the chemically modified amino acids, the calculated lipophilicity values for the standard amino acids were compared to those used in the hydrophobicity scales of Kyte-Doolittle [57] and Black-Mould [58]. With the exception of amino acids having pi-conjugated aromatic side chains, our predicted lipophilicity values for natural amino acid side chains were consistent with those used in these existing scales. It should be noted that conventional measures of lipophilicity primarily reflect the strength of hydrophobic interactions. However, they are also affected by polar interactions [59] because the model lipid used to determine partition coefficients (i.e. logP values) is octanol, which has two distinct functional groups: a hydrophobic (non-polar) alkyl chain and a hydrophilic (polar) hydroxyl group. We suggest that the inconsistency between our scale and those presented previously with respect to the lipophilicity values for aromatic amino acids (and tryptophan in particular) is because these molecules interact preferentially with the interfacial region of the lipid, in a way that is not primarily driven by classical hydrophobicity [60], [61].

**Table 1:** Normalized lipophilicity and solvent accessible surface area (SASA) values for natural and modified amino acids, assuming maximal side-chain exposure.

A.A.	LogP^a^	SASA^b^
Ala	0.015	65.85
Arg^+^	−0.62	200.62
Asn	−0.18	128.88
Asp^−^	−0.51	112.53
Cys^c^	0.068	99.87
Gln	−0.14	148.60
Glu^−^	−0.47	146.78
Gly^d^	0.00	0.00
His^e^	−0.060	157.99
Ile	0.38	157.72
Leu	0.19	166.64
Lys^+^	−0.366	195.16
Met	0.096	166.80
Phe	0.26	188.38
Pro^f^	0.25	105.72
Ser	−0.083	82.22
Thr	−0.015	114.60
Trp	0.28	229.66
Tyr	0.19	214.65
Val	0.20	125.98
Ack^g^	0.012	263.09
Cdr^+^ h	−0.49	336.44
Kyw^i^	0.10	236.08
Mee^j^	−0.012	182.28

a Scaled parameter = (raw parameters+3.800)/6.457, where the value of 3.800 is the raw parameter value for logP(Arg^+^) and the value of 6.457 is the sum of the absolute values of two raw parameters, logP(Arg^+^) and logP(Ile).

The normalized parameter values are equal to the scaled parameter values minus the scaled parameter value for logP(Gly).

b The SASA of amino acid (X) is the exposed solvent-accessible surface area of its side chain in the tripeptide Gly-X-Gly, assuming that ψ = φ = 180°.

C_α_ was not considered to be part of the side chain of X.

c Oxidized cysteine (Cys) has 50% of the lipophilicity of cystine (Css), i.e. the oxidized dimer of cysteine.

logP(Cys) = [logP(Css)]/2, where logP(Css) = logP(CH_2_-S-S-CH_2_).

d log(Gly) = log(H).

e At pH = 7.4, histidine exists in protonated (His^+^) and unprotonated forms (His^0^) with mole fractions of 11.2% and 88.8%, respectively.

logP(His) = logP(His^+^)⋅mole fraction (His^+^)+logP(His^0^)⋅mole fraction (His^0^).

f log(Pro) = log(CH_2_-CH_2_-CH_2_-N).

g Ack: acetylated lysine.

h Cdr^+^: modified arginine with 1,2-cyclohexanedione.

i Kyw: kynurenine, a metabolite of tryptophan.

j Mee: methyl-δ-glutamate.

#### Lipophilicity parameters (Ls and 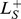) and the lipophilic moment (L_M_)

The value of the lipophilicity scalar for a given peptide depends on the exposed surface area of its amino acid side chains and their lipophilicity scale values. Two lipophilicity-related scalar quantities were calculated: the total lipophilicity (L_S_) and the exclusive lipophilicity 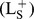. The total lipophilicity (L_S_) is based on the lipophilicity values of every residue in the peptide, regardless of their propensity for participating in lipophilic interactions. Conversely, the exclusive lipophilicity 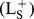 is based only on the lipophilicity values of residues that interact favorably with lipids. The total lipophilicity (L_S_) is thus proportional to the difference between the peptide's lipophilic and lipophobic surface areas, whereas the exclusive lipophilicity 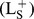 is proportional to the lipophilic surface area alone.

**Figure 5:**
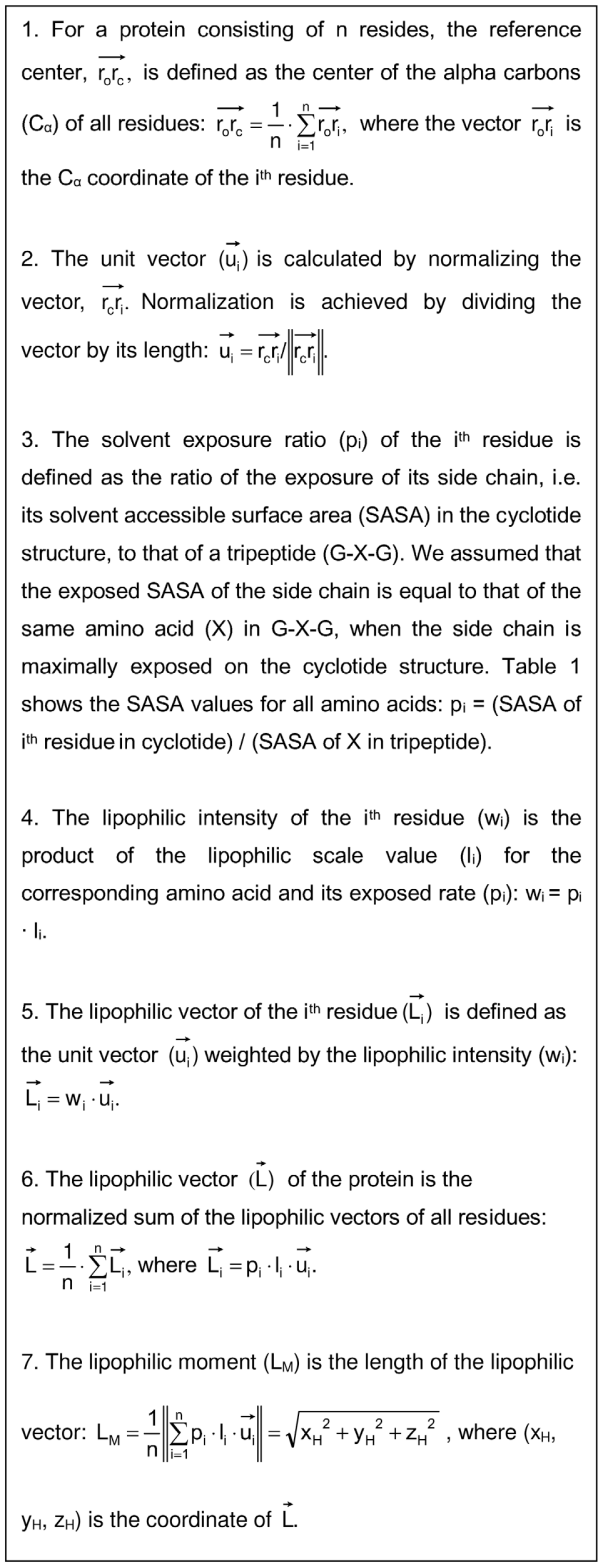
The calculation of lipophilic moment (L_M_).

The lipophilic moment (L_M_) is calculated from the sum of the lipophilic vectors of each residue in the protein, as shown in Figure 5. In brief, the lipophilic vector of a given residue is calculated based on its value on the lipophilicity scale and its position relative to the protein center. If the majority of the peptide's lipophilic residues are positioned on one side of molecular surface, and its lipophobic residues are positioned on the opposite side, its overall distribution of lipophilicity will be asymmetric. The greater the asymmetry in the distribution of lipophilicity across the peptide's surface, the greater its lipophilic moment. Introducing lipophobic residues into a lipophilic cluster will decrease the lipophilic moment by increasing the symmetry of the lipophilicity distribution. The procedure used to calculate L_M_ is described in the Figure S2.

The affinity of the protein for the membrane surface is proportional to the contact area, provided that the contact surface does not contain lipophobic moieties. In conjunction with the lipophilic moment (L_M_), the exclusive lipophilicity 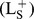 can be used to characterize the lipophilic strength of a membrane-active protein more accurately than is possible when using the total lipophilicity (L_S_) alone. This is because the lipophilic moment (L_M_) can be used to determine whether there is any significant lipophobic interruption of the contact surface, and the exclusive lipophilicity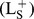 provides information on the proportion of the protein's total lipophilic surface area that lies within the contact region. Most biologically active cyclotides have large lipophilic moments, and their potency correlates positively with their exclusive lipophilicity 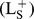. Lipophobic residues on the opposite side of the peptide surface to the contact region reduce its total lipophilicity (L_S_), but do not adversely affect its lipophilic interactions with membranes.

#### The positively charged surface area (E_S_) and the HBD amphipathic moment (E_M_)

**Figure 6:**
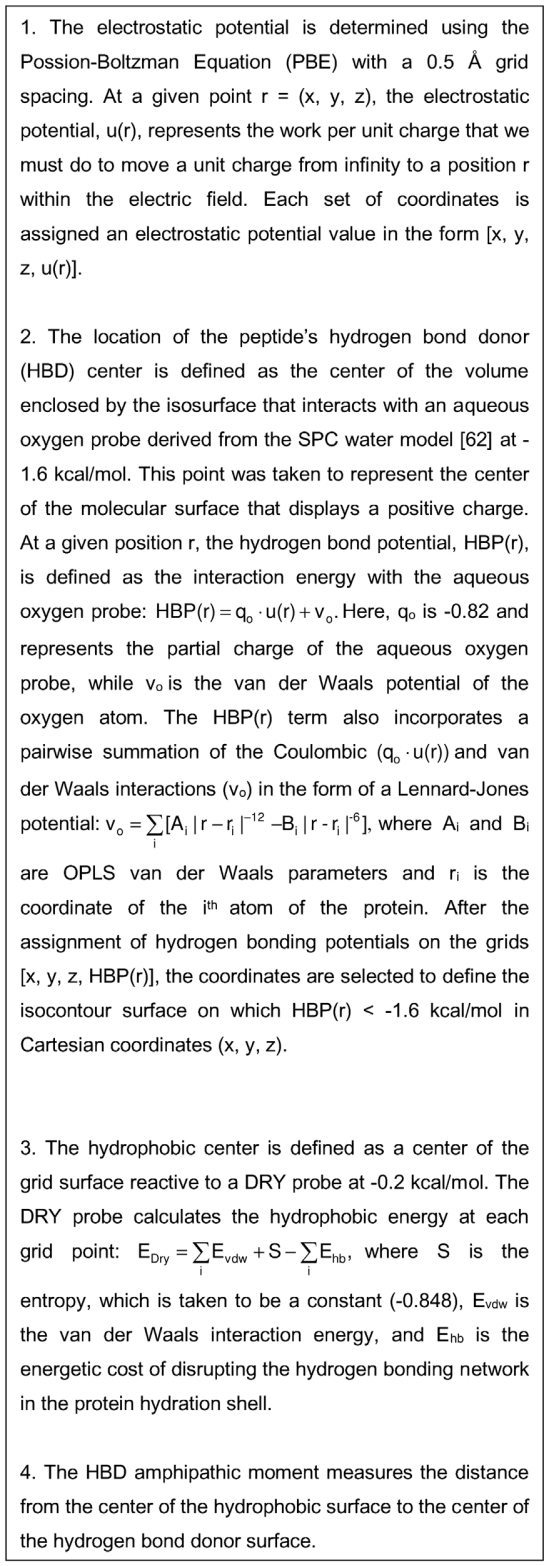
The calculation of the HBD amphipathic moment (E_M_).

The positively charged surface area of the peptide is defined as the hydrogen bond donor (HBD) surface (Figure 6). The HBD surface is determined from the electrostatic potential (*E*) of the van der Waals surface of the protein. It reflects the exposed surface area of the positively charged residues and their surrounding electrostatic environment (i.e. their proximity to other charged entities and their location on the interior or exterior of the protein).

The HBD amphipathic moment (E_M_) measures the imbalance in the electrostatic distribution between positively charged and hydrophobic (i.e. neutrally charged) surfaces. The magnitude of the moment is proportional to the distance between the hydrophobic center and the center of the positively charged surface. The moment is maximized in cases where the positively charged surfaces are localized on one side of the protein's surface and the hydrophobic surfaces are localized on the opposite side.

### Classification of cyclotides and their activities

The molecular descriptors discussed above were determined for every modeled structure, and compared qualitatively with the reported activities of the corresponding cyclotides. Cytotoxic [13], [18], [22], [47], [48], [49], [50] and anthelmintic [20], [51] activity data were obtained from the literature. Table 2 summarizes the potency of cyclotides and shows their IC_50_ values.

**Table 2:** Labels used to refer to each cyclotide considered in this work and their activities against *H. contortus* and cells from the U-937GTB line.

Label	Proteins	*H. contortus* IC_50_ (µM)	U-937GTB IC_50_ (µM)	Ref
	**Möbius**			
M1	kB1	2.48	6.9	[18], [20]
M2	[G1K]-kB1^a^	0.5	-	[20]
M3	[L2K]-kB1^a^	6.2	-	[20]
M4	[P3K]-kB1^a^	>11.5	-	[20]
M5	[V4K]-kB1^a^	11.1	-	[20]
M6	[G6K]-kB1^a^	>11.5	-	[20]
M7	[E7K]-kB1^a^	>11.5	-	[20]
M8	[T8K]-kB1^a^	>11.5	-	[20]
M9	[V10K]-kB1^a^	>11.5	-	[20]
M10	[G11K]-kB1^a^	5.3	-	[20]
M11	[G12K]-kB1^a^	>11.5	-	[20]
M12	[T13K]-kB1^a^	2.8	-	[20]
M13	[N15K]-kB1^a^	>11.5	-	[20]
M14	[T16K]-kB1^a^	>11.5	-	[20]
M15	[P17K]-kB1^a^	3.6	-	[20]
M16	[G18K]-kB1^a^	1.1	-	[20]
M17	[T20K]-kB1^a^	0.9	-	[20]
M18	[T20K, G1K]-kB1^a^	0.4	-	[20]
M19	[T20K, S22K]-kB1^a^	0.7	-	[20]
M20	[T20K, N29K]-kB1^a^	0.4	-	[20]
M21	[T20K, N29K, G1K]-kB1^a^	0.2	-	[20]
M22	[S22K]-kB1^a^	2.3	-	[20]
M23	[W23K]-kB1^a^	>11.5	-	[20]
M24	[V25K]-kB1^a^	>11.5	-	[20]
M25	[T27K]-kB1^a^	1.6	-	[20]
M26	[R28K]-kB1^a^	3.9	-	[20]
M27	[N29K]-kB1^a^	0.4	-	[20]
M28	kB2	1.59	2.6	[18], [51]
M29	kB6	0.87	-	[51]
M30	kB7	6.29	29	[18], [51]
M31	kB13	-	3.8	[18]
M32	cyH3	0.85	-	[51]
M33	cyO14	0.41	-	[51]
M34	cyO15	0.38	-	[51]
M35	cyO16	0.27	-	[51]
M36	cyO24	1.74	-	[51]
M37	vaby A	-	7.6	[47]
M38	vaby D	-	2.8	[47]
M39	varv A	1.13	8.2	[18], [51]
M40	[Cdr]-varv A	-	9.1	[18]
M41	[Cdr, Mee]-varv A	-	46	[18]
M42	[Mee]-varv A	-	34	[18]
M43	varv E	0.9	4	[49], [51]
M44	varv F	-	7.1	[13]
M45	vibi D	-	>30	[49]
	**Bracelet**			
B1	circulin A	-	-	-
B2	circulin B	-	-	-
B3	cyO1	2.82	-	[51]
B4	cyO2	0.12	1.03	[18], [51]
B5	[Cdr]-cyO2	-	0.95	[22]
B6	[Cdr, Ack]-cyO2	-	5.1	[22]
B7	[Mee]-cyO2	0.76	36	[22], [51]
B8	[Ack]-cyO2	2.3	2.3	[22], [51]
B9	[Kyw]-cyO2	-	5.2	[18]
B10	cyO3	0.21	-	[51]
B11	cyO8	0.24	-	[51]
B12	cyO13	0.21	-	[51]
B13	cyO19	-	0.52	[18]
B14	cyY4	2.01	-	[51]
B15	cyY5	2.28	-	[51]
B16	psyle E	-	0.76	[48]
B17	vhl-1	2.06	-	[51]
B18	vibi E	-	3.2	[49]
B19	vibi G	-	0.96	[49]
B20	vibi H	-	1.6	[49]
B21	vitri A	-	0.6	[50]
B22	vodo O	-	3.2	[18]
	**Hybrid**			
H1	[W23K, P24N, V25K]-kB1^a^	-	-	-
H2	[P24K]-kB1^a^	-	-	[20]
H3	[P24D, V25K]-kB1^a^	-	-	-
H4	kB8	-	18	[18]
H5	psyle A	-	2	[48]
H6	tricyclon A	-	-	-
	**Linear**			
L1	psyle C	-	3.5	[48]
L2	violacin A	-	-	-

a The first residue in the sequence of kalata B1 is defined relative to the C-terminal amino acid involved in the biosynthetic ligation in loop 6. (See Figure 3).

#### Physicochemical distribution of cyclotides

**Figure 7:**
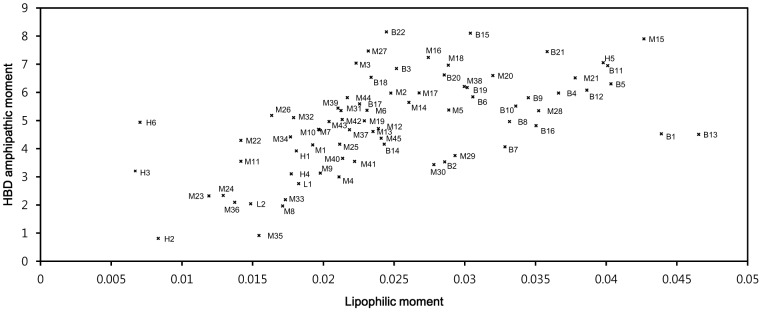
Moment plot (L_M_, E_M_) for the studied cyclotides. The cyclotides, labeled according to their subfamily (M for Möbius, B for bracelet, H for hybrid) and with their unique numbers, were plotted on a map based on their lipophilic moments (L_M_) and HBD amphipathic moments (E_M_). They cluster into two groups based on their moments: one group has low moments and exhibits low activity while members of the other group have high moments and exhibit moderate or high activity. The synthetic hybrid cyclotides, [W23K, P24N, V25K]-kB1 (H1), [P24K]-kB1 (H2) and [P24D, V25K]-kB1 (H3) cluster in low moment group. The activity of the cyclotides H1 and H3 against the U937GTB cell line and *H. contortus* is not known, but they are reported to be inactive against human type A erythrocytes [30].

The cyclotides clustered closely with similar sequences and levels of biological activity when their lipophilic (L_M_) and HBD amphipathic (E_M_) moments were plotted against one-another (Figure 7). Most bracelet cyclotides have larger moments than those from the Möbius and hybrid subfamilies. Moreover, Möbius cyclotides generally have higher moments than hybrid cyclotides; the exceptions are the Möbius cyclotides that have mutations attenuating lipophilicity in loop 5. When combined with activity data, the low moment group coincides with cyclotides that we classify as having low activity, and cyclotides having moderate or high moments have moderate to high activity. The relative activity was calculated by comparing IC_50_ values in the cytotoxicity and anthelmintic activity assays to the IC_50_ of kB1. Cyclotides with ratios (IC_50_ of cyclotide/IC_50_ of kB1) <0.2 were considered being highly active; ratios between 0.2 and 1 as moderately active; and ratios >1 (i.e. less active than kB1) as being low activity cyclotides.

**Figure 8:**
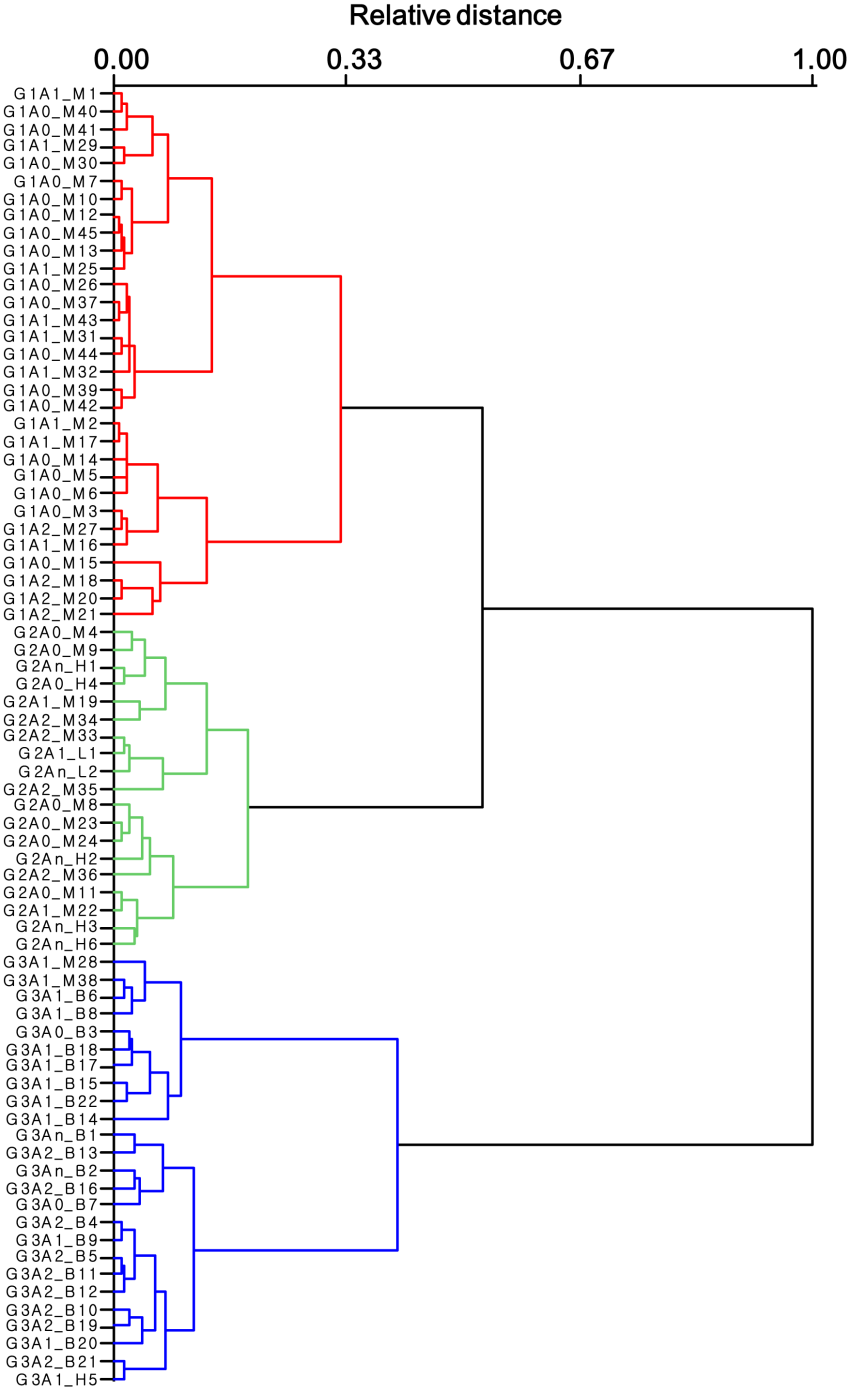
A dendrogram showing the hierarchical clustering of 75 cyclotides based on their molecular descriptors. The descriptors considered include the lipophilic moment (L_M_), exclusive lipophilicity 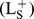, positively charged surface area (E_S_) and HBD amphipathic moment (E_M_). The left column shows the cyclotides' labels, which indicate the subgroup to which they belong (indicated by the letter ‘G’), relative activity (indicated by the letter ‘A’) and a subfamily-specific letter (M for Möbius, B for bracelet, H for hybrid and L for linear). The relative distance measures the physicochemical dissimilarity between cyclotides in respect to the normalized scale. The relative activity was calculated with respect to the cytotoxicity of kB1, and three levels of activity were defined: A0 for peptides whose relative activity ranges of >1.0, A1 for those with relative activities between 0.2 and 1.0, and A2 for those with relative activities of <0.2. Those with unknown activity were labeled An. Aside from [Mee]-cyO2 and varv A, all of the cyclotides were assigned to the same activity groups for both cytotoxicity and anthelmintic activity. The cyclotide subfamilies clustered into different subgroups, with most of the Möbius cyclotides being in groups 1 (red) and 2 (green), and all of the bracelets being in group 3 (blue). Aside from psyle A (H5), all of the hybrid cyclotides clustered in group 2. Group 2 also contains some unusual cyclotides that exhibit high or intermediate activity, namely cyO14 (M33), cyO15 (M34), cyO16 (M35), [T20K, S22K]-kB1 (M19) and [S22K]-kB1 (M22). These cyclotides are all rich in lysines.

The trend was reinforced when all four of the new molecular descriptors (E_M_, E_S_, L_M_ and 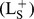) were used to analyze the relationships between the cyclotides: three distinct groups of different levels of activity emerged as demonstrated by the dendrogram in Figure 8. The cyclotide subfamilies clustered into different subgroups, with most of the Möbius cyclotides being in groups 1 and 2, and all of the bracelets being in group 3.

Aside from psyle A (H5), all hybrid cyclotides clustered in group 2. Two of them, kB8 (H4) and tricyclone A (H6), clustered with synthetic cyclotides that are known to be biologically inactive in antihelmintic assays, [W23K]-kB1 (M23), [V25K]-kB1 (M24) [20], or hemolytic assays, [W23K, P24N, V25K]-kB1 (H1), and [P24D, V25K]-kB1 (H3) [30]. These mutant cyclotides differ from native Möbius cyclotides, and resemble native hybrids, in that they have relatively low degree of lipophilicity in loop 5. These hybrid cyclotides exhibit a high degree of sequence identity with their Möbius counterparts in all domains other than loops 5 and 6. As such, it seems that reducing the lipophilicity of loop 5 decreases membrane activity of the Möbius cyclotide subfamily. We propose that this loop is important because it contributes significantly to the lipophilic contact area through which the Möbius cyclotide adsorbs to the membrane, and to the asymmetric distribution of lipophilicity within those peptides.

#### Loop lipophilicity and its impact on membrane orientation

**Figure 9:**
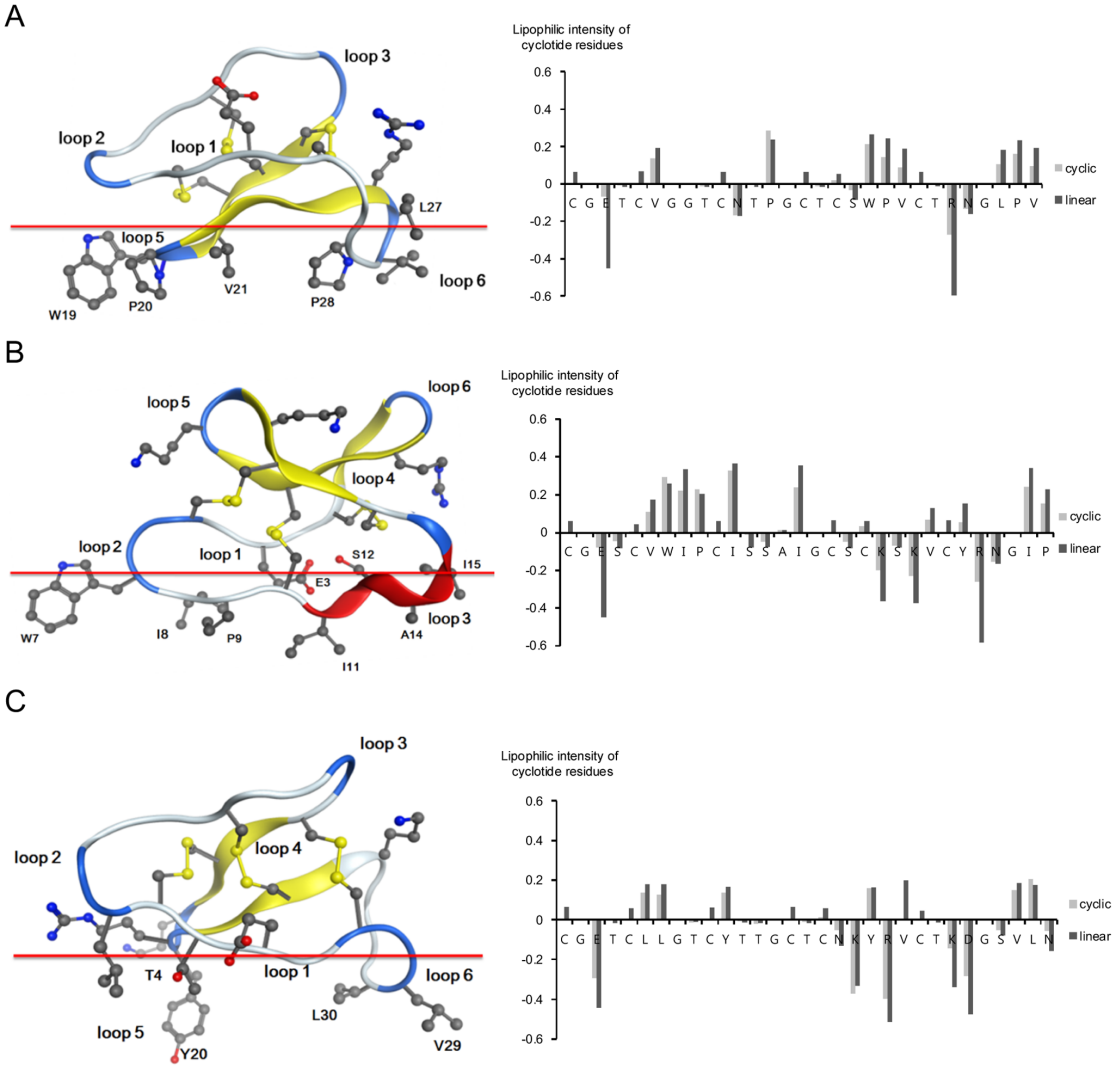
Predicted membrane binding modes (left) and lipophilicity profiles (right) for kalata B1 (A), cycloviolacin O2 (B) and kalata B8 (C) in their native cyclic conformations and in hypothetically unfolded conformations. The outer phosphate layer of a phospholipid membrane is represented by a red line in each case. Strained backbone regions with little structural mobility are indicated in blue. Backbone regions with a defined secondary structure are colored in yellow and red to indicate β-strands and α-helices, respectively. Only selected side chains are depicted, including those that form the hydrophobic patch, residues carrying positive charges, and the conserved glutamic acid in loop 1. The cyclotide structures were obtained from the PDB server; their PDB IDs are 1NB1 (kalata B1) [28], 2KNM (cycloviolacin O2) [33], and 2B38 (kalata B8) [16]. The lipophilicity profile of each residue was calculated for the natively folded cyclotides and for their unfolded conformations. The hypothetical unfolded cyclotide conformations were defined such that ψ = φ = 180°. It should be noted that in the natively folded conformations of kalata B1 (kB1) and cycloviolacin O2 (cyO2), the degree of solvent exposure for lipophilic residues is high while that for lipophobic residues is low. For example, in loop 2 of cyO2, the lipophilicity of Trp and Pro is higher in the native fold than in the unfolded conformation. Moreover, the lipophobicity values for polar residues are relatively high in natively folded kB8 compared to natively folded kB1 and cyO2. In contrast to the situation for kB1 and cyO2, the polar residues of kB8 exhibit similar lipophobicities in the native and unfolded conformations. Notably, the glutamic acid residue in loop 1 of kB8 participates in an internal hydrogen-bonding network within the core of the protein that effectively minimizes its SASA value. Consequently, it exhibits a much higher level of lipophobicity in the native fold than do the equivalent residues of kB1 and cyO2.

The cyclotide subfamilies interact with membranes in ways that reflect their differences in terms of the distribution of lipophilicity across the molecular surface. Due to the high levels of sequence similarity within cyclotide subfamilies and their very similar folds, cyclotides from the same subfamily generally have very similar spatial distributions of lipophilicity and therefore adopt very similar orientations when associated with membranes (Figure 3 and 9). Strongly lipophilic loops are typically buried in the interior of the membrane. This is consistent with the predictions of the “Orientations of Proteins in Membranes” (OPM) database for these peptides [63]. The Möbius and hybrid subfamilies adopt different orientations on the membrane because of their differences with respect to the lipophilicity of loop 5: in the Möbius cyclotides, this loop is rich in lipophilic residues but in the hybrids, it is relatively lipophobic. Consequently, whereas the Möbius cyclotides have large membrane-buried regions that include this loop, hybrid cyclotides cannot interact with membranes in this way. Loop 6 is the largest loop in all of the cyclotide subfamilies, and is the loop with the highest degree of sequence diversity within individual subfamilies. Therefore, the lipophilicity of this loop differs significantly between individual members of specific subfamilies. Even though it has negative lipophilicity values in many members of the Möbius and hybrid subfamilies, some residues from loop 6 may nevertheless become buried in the membrane.

The conformational constraints imposed by the cyclic cystine knot (CCK) motif increase the “lipophilic intensity” (see Figure 5) of the cyclotides by increasing the solvent-accessible surface area (SASA) of the lipophilic residues and reducing the SASA of lipophobic residues. The cystine knot is deeply buried in the protein core, and each pair of cystines fixes the end points of the loops in close proximity to one-another. Thus, rather than interacting directly with the membrane, the cystines force the backbones of the loops to curve outwards. Because of this knot structure, the lipophilic side-chains of cyclotides are generally oriented towards the exterior of the peptide in convex loops with a high rate of solvent accessible surface, despite this energetically unfavorable state. This combination of high lipophilicity and a convex backbone is illustrated in Figure 9, which shows loops 5 and 2 of the Möbius and bracelet subfamilies, respectively. Loop 5 and loop 2 are the most lipophilic loops in these subfamilies, and both contain aromatic residues in close proximity to a proline residue. In loop 5 of the Möbius subfamily, the aromatic residue (X^Ar^) is directly adjacent to a *cis*-proline (X^Ar^+*cis*-Pro). In loop 2 of the bracelet subfamily, the aromatic residue is separated from a *trans*-proline residue by a single intermediate residue (X) that has an alkyl side chain (X^Ar^+X+*trans*-Pro). In contrast to loop 5 of the Möbius cyclotides, in which the aromatic residue stacks on top of its neighboring proline, the aromatic side chain in loop 2 of the bracelet subfamily protrudes outwards and does not interact with its neighboring residues. This extraordinary degree of protrusion increases the solvent-accessible surface area of the aromatic side chain and therefore significantly increases the peptide's lipophilicity. Furthermore, the convexity of the backbone increases when the loop is constrained by the CCK motif. Figure 9 shows the lipophilic intensity of each residue in three representative cyclotides in two conformations: the native fold and a hypothetically unfolded conformation in which Φ = ψ = 180°. The lipophilicity values for most residues are comparable in both conformations, but there are some important exceptions.

### QSAR analysis

QSARs assume that the biological activity of a compound is dependent on its physicochemical properties. Such relationships can be illustrated quantitatively when a series of related compounds are considered. The complexity of QSAR models stems from a number of different factors. One of these is that the introduction of additional terms into the equation can easily increase the square correlation coefficient (r^2^) via a phenomenon that is known as overfitting. This is especially problematic if the new term represents a variable that conveys physicochemical information that is also present in one of the other terms used in the model. In addition, the inclusion of polynomial terms, products of multiple terms, or descriptors whose meaning is obscure can produce models that are very accurate but hard to interpret. We therefore aimed to develop models that use a relatively small number of easily interpreted descriptors (Table 3).

**Table 3:** Molecular descriptors used in the QSAR models.

Descriptors	Description
τ	The variable τ is a diagonal matrix whose diagonal elements (τ_ii_) consist of the spline terms of E_M_ and L_M_, The value of a diagonal element (τ_ii_) is zero if both moments of the i^th^ cyclotide are below those of the critical point. Otherwise, the value of τ_ii_ is one.
E_S_	The positively charged surface area
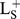	The exclusive lipophilicity. This quantity represents the sum of the lipophilic intensities of residues that have positive values on the lipophilicity scale and are located within regions that are, overall, attractive to lipids. In contrast, the quantity 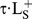 gives a measure of the lipophilicity of the lipophilic domain, i.e. the lipophilicity of all residues located on regions of the molecular surface that would be in contact with lipids when the protein is associated with a membrane.

**Figure 10:**
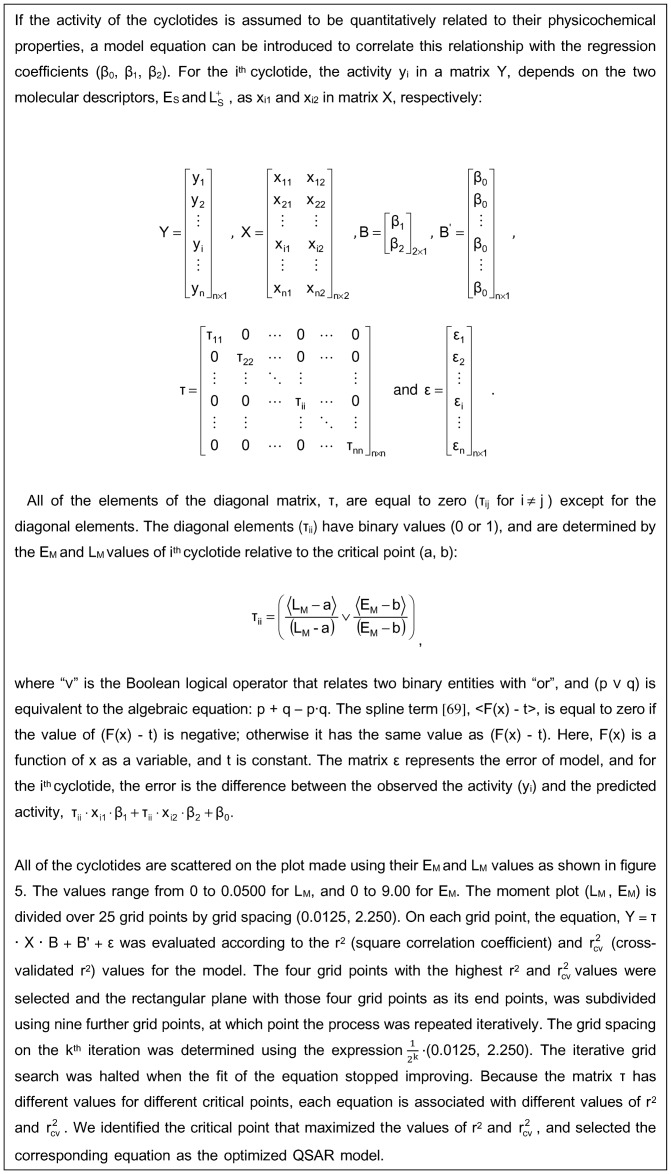
Generation of QSAR models.

The QSAR models were established using an equation that relates the activity of the studied cyclotides to the four physicochemical descriptors discussed in the preceding sections: the exclusive lipophilicity 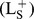, the positively charged surface area (E_S_), and the corresponding moments (L_M_ and E_M_) (Table 4). A process termed “dummy variable regression” was used to explain the observation that the cyclotides could be divided into two groups that had different moment values, with the high moment value group having high or intermediate activity and the low moment group having low activity. We hypothesized that relative activity only correlates with 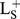 and E_S_ when the corresponding quantities were asymmetrically distributed over the molecular surface as a whole. The two groups of cyclotides were analyzed using a single regression equation featuring a dummy variable regressor, τ, which assigns a value of one to the first group and zero to the second. The hypothesis is embodied in the following regression equation: 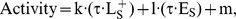 where k, l and m are constants. We then sought to define a critical point in terms of a pair of threshold moment values that would discriminate between cyclotides from the high and low activity groups, such that cyclotides in one group would have moment values above the critical point, while those in the other group would have moments below the critical point. A range of potential critical points was evaluated by considering the equations arising in each case; the optimal critical point was identified as that for which the correlation coefficient (r^2^) and cross-validated correlation coefficient 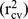 for the regression equation were maximized. The generation of the model and the introduction of the dummy variable τ is described in Figure 10.

**Table 4:** QSAR equations for activity against the U-937GTB line and *H. contortus*.

No	Equations	r^2^	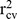	F	F^c^
1	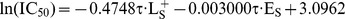	0.65	0.56	20.13	4.58
2	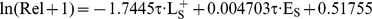	0.63	0.56	35.01	4.32

Equations 1 and 2 represent the QSAR models for activity against the U-937GTB line and *H. contortus*, respectively. The raw values of 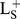 were transformed using the Box-Cox method, according to which y′ = y^λ^, where λ = 1.0 and −0.5 in equation 1 and 2, respectively. In equation 2, this transformation inverts the order of the 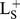 data set. Therefore, the negative sign of the coefficient reflects a positive correlation between 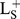 and the measured activity values. The F^c^ is the critical F-value at confidence level of 95%. “Rel” refers to the relative activity of the cyclotide in question compared to kalata B1.

**Figure 11:**
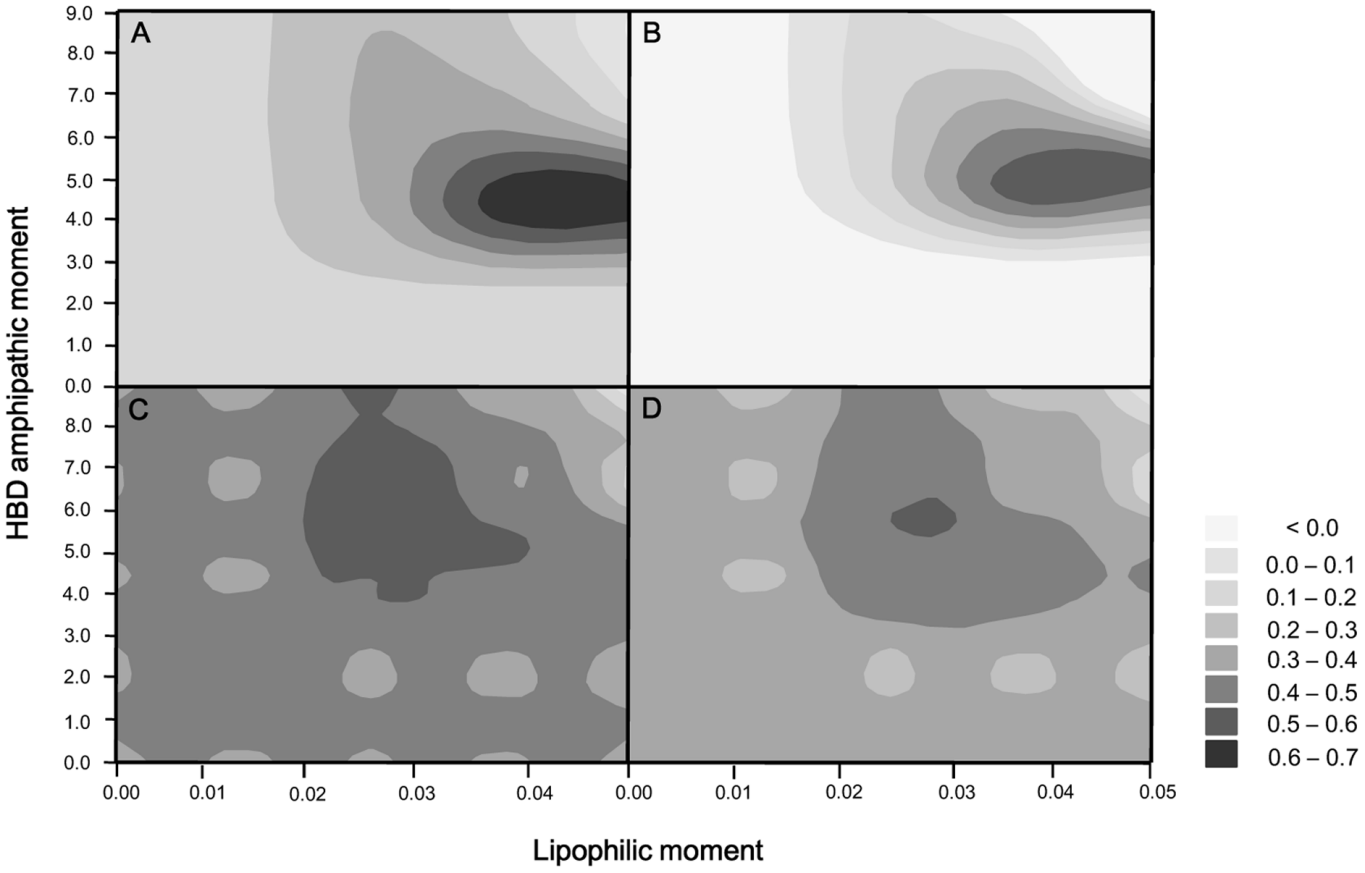
Statistical parameters for the moment plots (L_M_, E_M_). The grayscale values indicate the performance of the QSAR models based on the correlation coefficient (r^2^) and the cross-validated correlation coefficient 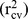 for the moment plot. Panels A and B show the values of r^2^ and 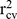 for the model describing cyclotide cytotoxicity against the lymphoma cell line, while panels C and D show the r^2^ and 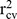 values for anthelmintic activity. Cyclotides whose moment values were below the critical point were placed in the low moment group, while those whose moments exceeded the critical point were placed in the high moment group. The critical points were determined to be (0.03750, 4.5000) and (0.02813, 5.6250) for activity against the U937GTB line and *H. contortus*, respectively.

The quality of the QSAR models could be improved to a remarkable extent by explaining the activity of cyclotides in a non-linear fashion using the exclusive lipophilicity 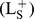 and positively charged surface area (E_S_) variables. This was done because we identified two groups of cyclotides with different moment values whose contact surfaces affect their activity in different ways, which we took to be indicative of a nonlinear correlation. Figure 11 shows the evaluation of model equations with these statistical parameters on the moment plot (L_M_, E_M_). The equation becomes a linear model when all of the diagonal components of τ are set to either 0 or 1. For all i, 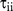 is 1 when a critical point is assigned on the grid at (0,0), and 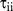 is 0, when a critical point is assigned on the grid at (0.050, 9.000). If a valid model (i.e. one with acceptable values of r^2^ and 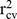) is obtained when the critical point is assumed to be located at (0, 0), the implication is that there is a positive correlation between activity and the surface area variables 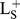 and E_S_ for all cyclotides. Conversely, if a valid model is obtained when the critical point is assumed to be located at (0.050, 9.000) on the grid, the implication is that there is no correlation between the activity and 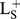 or E_S_ that is valid for all cyclotides. For cytotoxic activity, the critical point was determined to be (0.03750, 4.5000), and 7 of the 30 cyclotides for which the literature contains quantitative cytotoxicity data have moment values that place them below the critical point. For anthelmintic activity, the critical point was determined to be (0.02813, 5.6250); 19 of the 46 cyclotides for which the literature contains quantitative anthelmintic activity data have moments below this threshold.

When constructing the QSAR model for anthelmintic activity, the cyclotides were classified as non-active if their IC_50_ values exceeded the highest tested concentration, 11.5 µM [20]. The logarithm of the IC_50_ value is conventionally used to describe the potency of peptides. However, in this work we used a log(x+1) data transformation rather than a conventional ln(x) transformation in order to enable the inclusion of non-active cyclotides in the QSAR model. For cyclotides that were classified as active, the value of x was calculated based on their relative activity compared to kalata B1. Put simply, x is the ratio of the IC_50_ values of the cyclotide and kalata B1. Non-active cyclotides were assumed to have x values of 0, in which case the log(x+1) transformation also yields a value of 0 ( = log1). This prevents the transformed values from approaching negative infinity as they would if a simple ln(x) transformation were used but does not affect the normality of the data and keeps the variance relatively constant.

After inspecting the output of the initial QSAR models, outliers were removed from the data sets and new QSAR models were created. For the cytotoxicity QSAR model, the atypical linear cyclotide psyle C was removed. Similarly, cyO14, cyO15 and cyO16 were removed as outliers before generating the refined anthelmintic activity model. These cyclotides exhibit high cytotoxic activity despite having exceptionally low E_M_ and L_M_ moments, are rich in lysines, and stands out in when compared to other Möbius cyclotides. This discrepancy may indicate that these cyclotides exert their cytotoxic activity by different mechanisms to the other cytotoxic cyclotides. These three peptides have large positively charged surface areas that may form favorable electrostatic interactions with the membrane. In this context, it should be noted that a deep lipophilic patch is not an absolute prerequisite for membrane interaction [64]; it may be that these proteins adhere to the membrane surface due to electrostatic interactions with its charged phospholipid head groups and the moderately polar glyceryl/carbonyl groups just below them.

Our model for predicting cyclotide potency does not require any specific assumptions based on prior knowledge such as the location of PE binding sites or bioactive faces. Instead, it assumes that the potency of membrane-active peptides is determined by their affinity for membranes, which is in turn dictated by the size and heterogeneity of the contact surface. The location of the contact surface can be estimated by considering the orientation of the lipophilic vector; if the predicted contact surface features a large proportion of lipophobic side chains, the peptide would not be predicted to interact strongly with membranes. This hypothesis explained the observed activity of most of the studied cyclotides quite well, as indicated by the dendrogram shown in Figure 8. We suggest that those peptides whose activity is not well explained by this hypothesis exert their effects by some alternative mechanism. Recently, the first such specific cyclotide receptor interaction was reported: the binding of kalata B7 to the oxytocin receptor [65]. Is it possible that the current model can be used to study such specific, “classical”, protein receptor – peptide ligand interactions too? The answer is yes, because the fundamental principle of affinity is the same between protein-peptide interactions and protein-membrane interactions: the affinity increases with contact surface area. However, in the current case, the suitability of the model will depend heavily on rigidity and size of the peptide and the type of force mediating molecular interaction to the target. For extremely rigid peptides, such as cyclotides, in which there is no room for structural rearrangements upon docking into a receptor, it is clear that the full surface area that interacts with the target must be taken into account. The model could also be used to discover possible specific interactions: a peptide that clusters together with peptides of similar physicochemical properties but stands out in terms of activity indicates the presence of a specific interaction.

**Figure 12:**
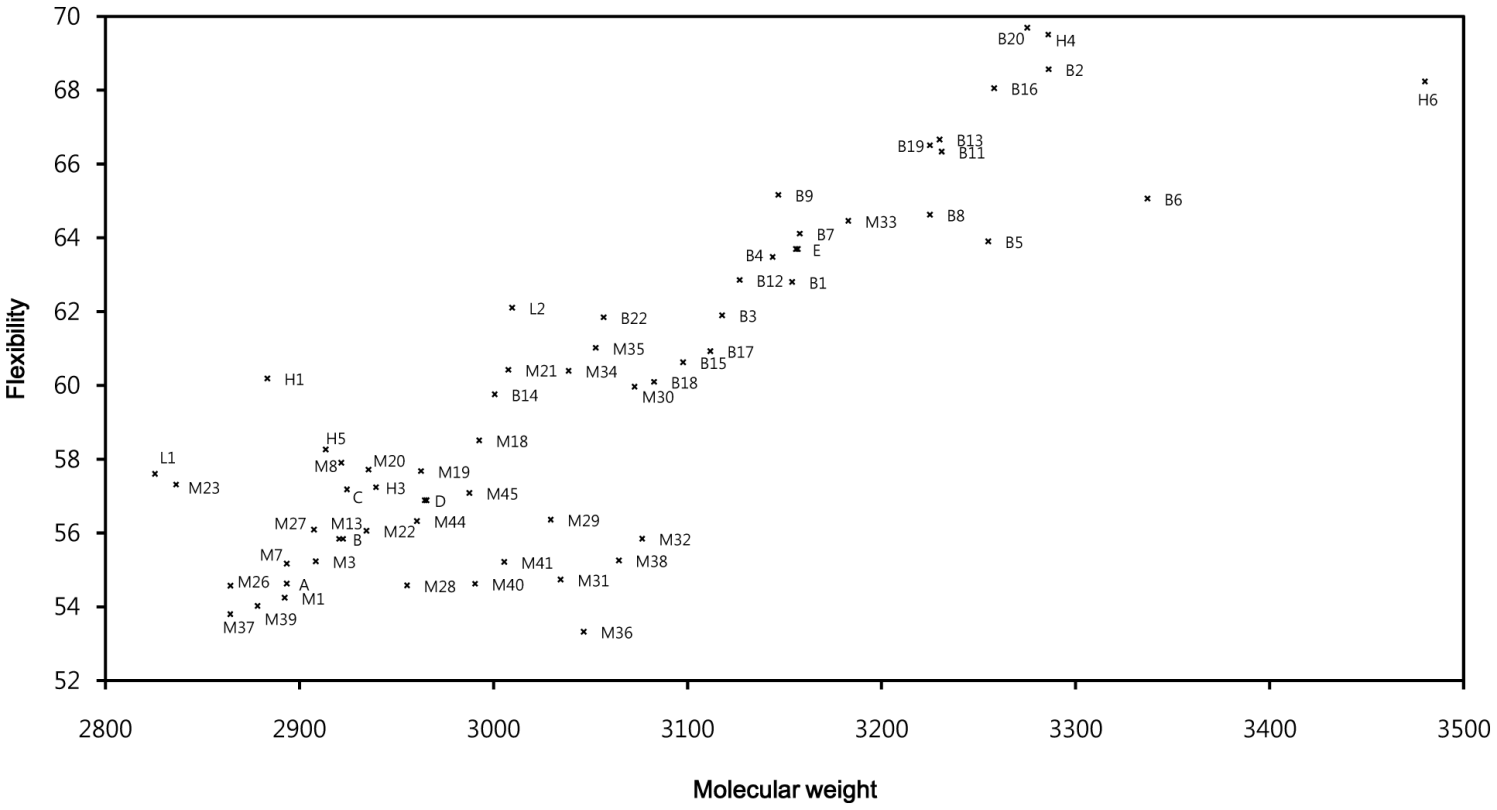
Chart of molecular weight and flexibility in cyclotides. This chart illustrates the correlation between flexibility and molecular weight. Points where multiple cyclotides overlap are indicated by the letters A-E; the cyclotides at these points are: A (M42, M43), B (M5, M9, M12, M14, M17, M24, M25), C (M4, H2), D (M2, M6, M10, M11, M16) and E (B10, B21). The linear cyclotide (L1 and L2) is relatively flexible since it lacks a cyclic backbone. The members of the hybrid cyclotide subfamily (H4, H6) have high molecular weights and flexibility values.

To facilitate the interpretation of the QSAR models, we chose to disregard molecular descriptors that exhibited any co-linearity with molecular size such as molecular weight and flexibility [66]. Because cyclotide activity correlates positively with the size of the membrane contact area, the use of descriptors that contain information on molecular size could potentially result in misleading causal interpretations. In particular, if it is assumed that the surface distribution of lipophilicity and high electrostatic potential do not vary, the potency of the molecules would increase with their size (in proportion to 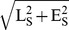). There is a positive linear correlation between flexibility and molecular size (r^2^ = 0.79) for molecules containing the CCK motif (Figure 12). The cyclic backbone and the disulfide bond network constrain the peptide's degree of freedom (which is proportional to flexibility), and the degree of freedom increases with the number of atoms (which is proportional to molecular weight). However, it has been reported that enhanced flexibility also reduces cyclotide activity due to reduced membrane adsorption [67] and stability [68].

The cytotoxicity and anthelmintic activity models both have correlation coefficients between 0.6 and 0.7. We believe that the main sources of error in the models relate to the fact that membrane-active proteins necessarily bind to membranes via non-specific interactions. For example, cyclotides drift over the fluid membrane surface, and can adopt a wide range of tilt angles as they do so. In addition, cyclotides can form oligomers on the membrane surface. Our model explains the potency of the cyclotides purely in terms of their physical contact with membranes. However, this approach implicitly assumes that the peptides interact as monomers and adopt a single static conformation with respect to the membrane surface. While the physical parameters (the lipophilic and HBD amphipathic surface areas and their distribution) used in this work were derived directly from the protein structure alone, their relevance for membrane binding is supported by the OPM database. Strong correlations have often been reported for QSAR models based on specific interactions such as ligand-protein interactions, but lower coefficients are reasonable for models based on non-specific interactions such as those that govern the binding of membrane-active proteins. Indeed, with one exception, no previous QSAR study on membrane-active peptides has yielded a regression coefficient in excess of 0.7 [23], [24], [26]. The sole exception is a study that used more than twenty molecular descriptors to obtain an r^2^ value of more than 0.9 [25].

## Conclusions

Cyclotides exert their biological effects via membrane disruption. We have presented a quantitative analysis of their structure-activity relationships based on two molecular properties that are important in membrane interactions: lipophilicity and an electrostatic property. Both of these properties were characterized using two dimensional quantities (a scalar and a moment), and their correlations with the peptides' biological activities were evaluated using one model equation. Our QSAR model suggests that there is a non-linear positive correlation between cyclotide potency and the (scalar) size of the molecular surface that interacts with the membrane. The nonlinearity is explained by the moments; a linear positive correlation is only observed for peptides whose lipophilic and electrostatic properties are unevenly distributed on the molecular surface. Furthermore, we qualitatively demonstrated that these molecular descriptors are useful in explaining how the physicochemical properties of cyclotides differ between subfamilies, and how these differences affect the orientation of cyclotides on the membrane and their membrane activity. Our results also illustrate how the cyclic cystine knot (CCK) motif forces cyclotides to adopt a conformation that enhances their lipophilicity.

The approach presented herein could be extended to cover other membrane-active proteins that contain non-standard amino acids. This is because lipophilicity and electronic charge data are readily determined for all amino acids, including non-standard ones. In addition, it can provide clear guidelines for drug design by predicting how specific changes will affect the surface properties of the peptide (i.e. the number of surface-accessible lipophilic and positively charged residues) and their relative distribution (i.e. their location on the molecular surface relative to other residues with similar properties).

## Supporting Information

Figure S1 RMSD distribution and solvent surface area rate of cyclotides' NMR ensembles.
(PDF)

Figure S2Example of calculation procedure of lipophilic parameters (L_M_ and 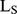).(PDF)
