# Supplementary material for: Cyclotide Structure–Activity Relationships: Qualitative and Quantitative Approaches Linking Cytotoxic and Anthelmintic Activity to the Clustering of Physicochemical Forces
Source: PLoS One. 2014 Mar 28;9(3):e91430. doi: 10.1371/journal.pone.0091430 (PMC3969350; doi:10.1371/journal.pone.0091430)
Supplement: Figure S1 — RMSD distribution and solvent surface area rate of cyclotides' NMR ensembles. (PDF) [file pone.0091430.s001.pdf]

RMSD distribution and solvent surface area rate of cyclotides' NMR ensembles.

Figure S1.A

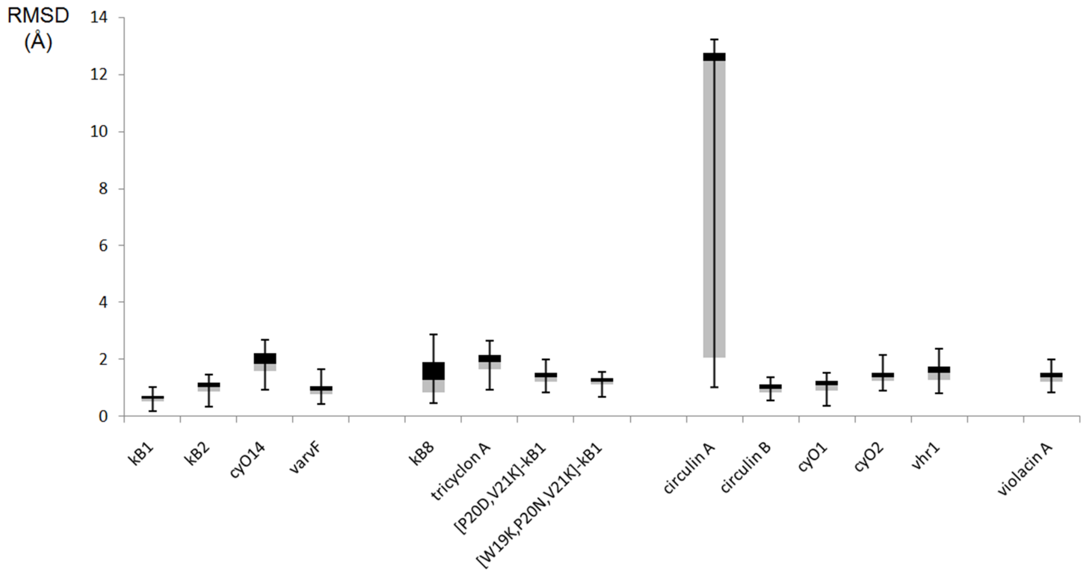

RMSD distribution of NMR ensemble in cyclotides. The box plot in Figure S1.A shows RMSD distribution of NMR ensembles for cyclotides used as templates. The gray colored box ranges from the first quartile to the median, and the black colored box ranges from the median to the third quartile. The lower and upper error bars represent the minimum and maximum RMSD, respectively. For each cyclotide, RMSD was calculated in accordance with the carbon and heteroatoms constructing the protein backbone and side chains. For each cyclotide, RMSD values were collected from pairwise RMSD matrix if the pairwise matrix (M_{ij}) satisfies: M_{ij}, where $i < j$. Except from circulin A, the average of RMSD of cyclotide structures do not exceed 2.0 Å: circulin A was not used as template for any cyclotide sequence in this work.

Figure S1.B

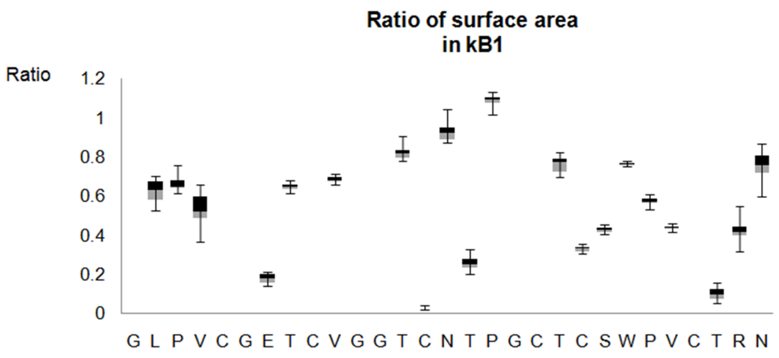

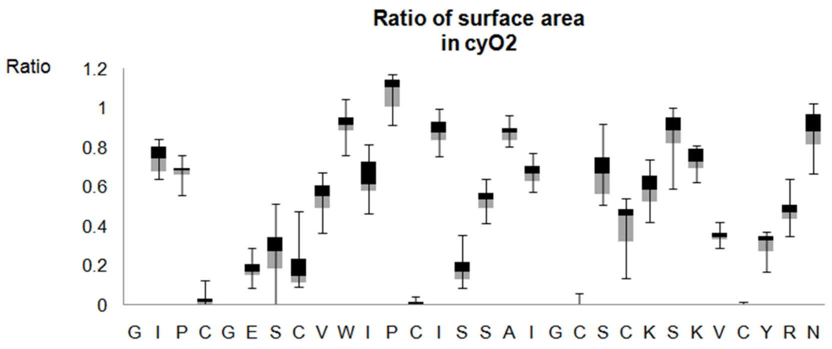

Figure S1.B shows the variations in the ratios of exposed surface areas for each amino acid residue of the cyclotides kalata B1 and cycloviolacin O2. (See Figure 5 for the definition of solvent exposure ratio.) The gray colored box ranges from the first quartile to the median, and the black colored box ranges from the median to the third quartile. The lower and upper error bars represent the minimum and maximum solvent exposure ratio, respectively. As expected from the similarities in backbone and side chain RMSD the ratios are more or less constant, which demonstrate that molecular surfaces within the NMR ensembles are not influenced by conformational changes.
