# Supplementary material for: Cyclotide Structure–Activity Relationships: Qualitative and Quantitative Approaches Linking Cytotoxic and Anthelmintic Activity to the Clustering of Physicochemical Forces
Source: PLoS One. 2014 Mar 28;9(3):e91430. doi: 10.1371/journal.pone.0091430 (PMC3969350; doi:10.1371/journal.pone.0091430)

Example of calculation procedure of lipophilic parameters (L_M and L_s)

This example shows the effect of an amino acid shift on the protein's lipophilic properties. The calculation of lipophilic moments and overall lipophilicity is exemplified for three cyclic octapeptides. The common peptide sequence is cyclo(Ile-Gly-Cys-Gly-X-Gly-Cys-Gly), in which the 5th residue, X, is a mutation site representing one of Ile, Gly or Lys. For simplicity, we assume that all of the C_α atoms lie in the xy-plane (z = 0), that the side chains of Ile, Gly and Lys are 50 % exposed, and that those of the two Cys residues are fully buried inside the protein core and therefore have exposed rates of 0 %. We further assume that the angles between the lines connecting each C_α to the center of the protein (which is defined by the positions of the C_α atoms) are 45°, i.e. that for i = 1,...,7, $\angle r_i r_c r_{i+1} = 45°$.

1. The location of the reference center ($\overrightarrow{r_o r_c}$) is determined by averaging the vectors pointing from the origin, r_o= (0, 0, 0) to the C_α atoms of n residues. (Figure S2.A)

$$\overrightarrow{r_o r_c} = \frac{1}{8} \cdot \sum_{i=1}^{8} \overrightarrow{r_o r_i},$$

2. For each ith residue, the unit vector ($\overrightarrow{u_i}$) is calculated by normalizing the vector pointing from the reference center to the C_α of the ith residue. Normalization is achieved by dividing this vector by its length.

$$\overrightarrow{u_i} = \overrightarrow{r_c r_i} / \left\| \overrightarrow{r_c r_i} \right\|$$

After normalization, the vector's length is 1 and its orientation is preserved. After the introduction of the r_c, the x, y and z axes are transformed into x', y' and z'. (Figure S2.B)

3. The exposed rate (p_i) of the ith residue is defined as the ratio of the solvent-accessible surface area (SASA) of its side chain in the peptide structure to the SASA for the side chain of the same amino acid (X') in the tripeptide (G-X'-G). We assume that the exposed SASA of the side chain is equal to SASA of the same amino acid (X') in the tripeptide (G-X'-G) if the side chain is fully exposed in the given conformation. The radius of the water probe was set to 1.4 Å. In this example, the exposed rates for the two cysteines and two glycines are zero while those for the other residues are 0.5. (Figure S2.C)

$$p_i = (\text{SASA of exposed } i^{th} \text{ side chain}) / (\text{SASA of fully exposed } i^{th} \text{ side chain})$$

4. The lipophilic intensity of the ith residue (w_i) is the product of the lipophilic index value (l_i) of the corresponding amino acid and its exposed rate (p_i) in the protein structure. In this example, the lipophilic intensities of the two cysteines and two glycines are zero because their exposed rates are zero. The lipophilic intensities of the other residues, isoleucine and lysine, are 0.19 (= 0.38 × 0.5) and -0.185 (= -0.37 × 0.5), respectively.

$$w_i = p_i \cdot l_i$$

5. The lipophilic vector of the ith residue ($\overrightarrow{L_i}$) is defined as the unit vector ($\overrightarrow{u_i}$) weighted with the

lipophilic intensity (w_i).

$$\overrightarrow{L_i} = w_i \cdot \overrightarrow{u_i}.$$

6. The lipophilic vector $(\vec{L})$ of the protein is the normalized sum of the lipophilic vectors of all residues. The sum is normalized against the total number of the residues. Let (x_H, y_H, z_H) be the coordinate of $\vec{L}$.

$$\vec{L} = \frac{1}{8} \cdot \sum_{i=1}^{8} \overrightarrow{L_i} = \frac{1}{8} \cdot \sum_{i=1}^{8} w_i \cdot \overrightarrow{u_i} = \frac{1}{8} \cdot \sum_{i=1}^{8} p_i l_i \overrightarrow{u_i} = (x_H, y_H, z_H)$$

7. The lipophilic moment (L_M) is the length of lipophilic vector.

$$L_M = \frac{1}{8} \left\| \sum_{i=1}^{8} w_i \cdot \overrightarrow{u_i} \right\| = \sqrt{x_H{}^2 + y_H{}^2 + z_H{}^2}$$

8. The overall lipophilicity is the sum of the lipophilic intensities of all residues and does not account for their relative orientation.

$$L_S = \sum_{i=1}^{8} w_i$$

The calculated lipophilic properties of the selected example peptides.

Lipophilic property	Mutation residue	Value	Details of calculation
Lipophilic vector	Lys	(0, 0.0469, 0)	$\frac{1}{8} \cdot [0.38 \times 0.5 \times (0,1,0) + (-0.37) \times 0.5 \times (0,-1,0)]$
	Gly	(0, 0.0238, 0)	$\frac{1}{8} \cdot [0.38 \times 0.5 \times (0,1,0) + 0 \times 0.5 \times (0,-1,0)]$
	Ile	(0, 0, 0)	$\frac{1}{8} \cdot [0.38 \times 0.5 \times (0,1,0) + 0.38 \times 0.5 \times (0,-1,0)]$
Lipophilic moment	Lys	0.0469	$\sqrt{0^2 + 0.0469^2 + 0^2}$
	Gly	0.0238	$\sqrt{0^2 + 0.0238^2 + 0^2}$
	Ile	0	$\sqrt{0^2 + 0^2 + 0^2}$
Overall lipophilicity	Lys	0.005	0.38 × 0.5 + (-0.37) × 0.5
	Gly	0.19	0.38 × 0.5 + 0 × 0.5
	Ile	0.38	0.38 × 0.5 + 0.38 × 0.5

The mutated amino acid is the 5th residue (X) in the sequence of the following cyclic peptide: cyclo(Ile-Gly-Cys-Gly-X-Gly-Cys-Gly). The details of the calculation for the lipophilic vector and overall lipophilicity only show two residues explicitly. Specifically, the first and second terms shown in the expressions relate to the first residue in the sequence and the fifth (i.e. the mutation site), respectively. This is because the lipophilic intensity (w_i) of the ith residue is zero for i = 2, 3, 4, 6, 7, 8, since either p_i = 0 or l_i = 0 for the glycines and the fully buried cysteines, respectively. It should be noted that making Ile the fifth residue doubles the overall lipophilicity compared to that obtained when using Gly as the fifth residue, but gives an overall lipophilic moment of zero. Also, the introduction of lysine in the fifth position almost doubles the lipophilic moment, but gives an overall lipophilicity of almost zero. The fifth residue is located almost directly opposite the first residue relative to the reference point.

Figure S2.A

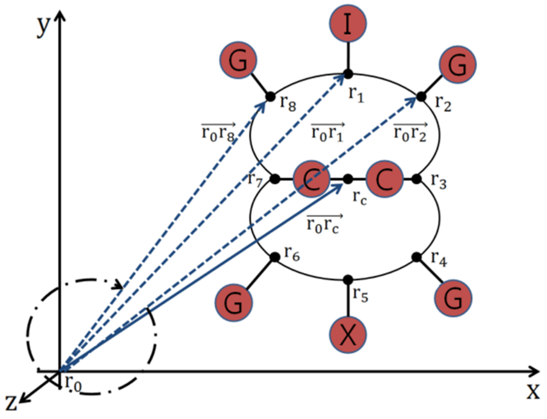

Figure S2.B

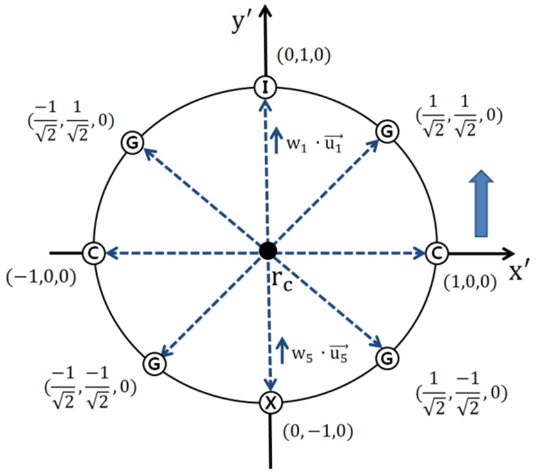

Figure S2.C

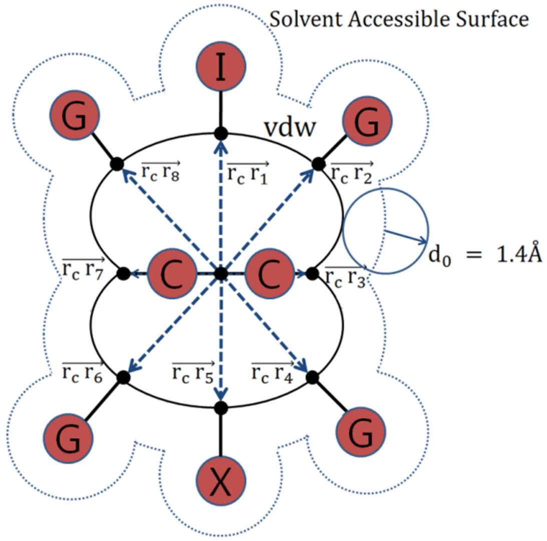

Supplement: Figure S2 — Example of calculation procedure of lipophilic parameters (LM and ). (PDF) [file pone.0091430.s002.pdf]
